# Concurrent chemoradiotherapy with conventional fractionated radiotherapy and low-dose daily cisplatin plus weekly docetaxel for T2N0 glottic cancer

**DOI:** 10.1186/s13014-016-0741-4

**Published:** 2017-02-20

**Authors:** Jun-ichi Saitoh, Katsuyuki Shirai, Masumi Imaeda, Atsushi Musha, Takanori Abe, Masato Shino, Yukihiro Takayasu, Katsumasa Takahashi, Kazuaki Chikamatsu, Takashi Nakano

**Affiliations:** 1 Department of Radiation Oncology, Gunma University Graduate School of Medicine, 3-39-22 Showa-machi, Maebashi, Gunma 371-8511 Japan; 2 Gunma University Heavy Ion Medical Center, Maebashi, Gunma Japan; 3 Department of Otolaryngology-Head & Neck Surgery, Gunma University Graduate School of Medicine, Maebashi, Gunma Japan

**Keywords:** Laryngeal cancer, Radiation therapy, Chemotherapy, Voice preservation

## Abstract

**Background:**

To assess the efficacy of concurrent chemoradiotherapy (CCRT) with daily low-dose cisplatin (CDDP) plus weekly docetaxel (DTX) for patients with T2N0 glottic cancer.

**Methods:**

Between January 2004 and December 2013, 62 treatment-naive patients with histologically proven T2N0 glottic cancer were treated with concurrent chemoradiotherapy. Radiation therapy (RT; 2 Gy daily fractions up to a total dose of 66 Gy) was administered in combination with daily low-dose CDDP (6 mg/m^2^, five times a week), plus weekly DTX (10 mg/m^2^) for up to 4 weeks from the commencement of RT.

**Results:**

Median duration of follow-up was 70 months. The actuarial 3-year and 5-year overall survival rates were 95% and 93%. The 3-year and 5-year cause-specific survival rates were both 100%. The actuarial 3-year and 5-year local control rates were 94% and 94%, respectively. Hematologic toxicity (neutoropenia of severity ≥ Grade 3) was observed in 8% of the patients, and non-hematologic toxicity (radiation mucositis of severity ≥ Grade 3) developed in one patient (2%). Radiation dermatitis of severity ≥ Grade 3 and laryngeal necrosis developed in one patient.

**Conclusion:**

CCRT with weekly DTX and low-dose CDDP appears to be a practical and safe modality and is expected to improve local control.

**Trial registration:**

UMIN000025046. Registered 1 October 2015, retrospectively registered.

## Background

A key focus in the management of laryngeal cancer is to improve survival with functional preservation. In particular, radiation therapy (RT) at an early stage is an important strategy for voice preservation in these patients. The local control rate with RT alone for T1 glottic cancer is of the order of 80 to 95%, whereas that for T2 disease is 70 to 80% [1–6]. These results suggest that conventional RT alone may not achieve satisfactory local control in patients with T2 disease.

Several approaches have been attempted to improve the local control of T2 glottic cancer, including hyperfractionated radiotherapy (HFRT) and accelerated fractionated radiotherapy (AFRT). Some earlier reports on altered fractionated radiotherapy indicated improved local control in patients with T2 glottic cancer. However, two recent prospective randomized trials of conventional fractionated radiotherapy (CFRT) versus HFRT/AFRT did not show a statistically significant difference in outcomes between the two groups. The 5-year local control rate was reported to be in the range of 70 to 78% [7, 8].

Concurrent chemoradiotherapy (CCRT) is another approach to improve local control. Docetaxel (DTX) was shown to enhance radiation sensitivity in pre-clinical studies [9–11]. The potential mechanisms of this radiation-enhancing effect of DTX include cell cycle synchronization, apoptosis, and reoxygenation. Several pre-clinical studies have suggested that cisplatin (CDDP) also modulates radiation cell damage effectively [12, 13]. At our institute, the CCRT regimen used for patients with T2 glottic cancer includes low-dose daily administration of CDDP plus weekly administration of DTX. Initial results were first reported in 2006 [14]. Five-year disease-free survival in patients who received RT alone (*n* = 25) and those who received CCRT (*n* = 25) were 70.9 and 91.8%, respectively. CCRT became a standard treatment option for patients with T2N0 laryngeal cancer with adequate renal and liver function and a reasonably good performance status.

In this series, an evaluation of the long-term follow-up outcomes of CCRT in 62 consecutive patients with T2N0 glottic cancer are presented.

## Methods

### Patients

Between January 2004 and December 2013, 62 (59 men and three women) patients with histopathologically confirmed T2N0 glottic cancer were treated with CCRT at our institute. The regimen consisted of RT plus daily administration of low-dose CDDP and weekly DTX. Median age of patients was 71 years (range, 51–88 years). The main eligibility criteria for CCRT were as follows: (i) patients with previously untreated, histologically proven, T2N0M0 glottic cancer; (ii) no previous history of regional RT; (iii) Eastern Cooperative Oncology Group performance status ≤2; (iv) sufficient organ function for chemoradiotherapy.

Sixty one patients had squamous cell carcinoma, and one patient had undifferentiated carcinoma. The distribution of the primary sites was as follows: supraglottic extension, 58 patients; subglottic extension, two patients; supraglottic and subglottic extension, two patients. Written informed consent to the treatment was obtained from all patients prior to commencement of the treatment.

### Treatment

The RT dosage schedule was 2 Gy per fraction, once a day up to a total dose of 66 Gy in 33 fractions, over a period of 7 weeks, in principle. The gross tumor volume (GTV) was assessed by laryngeal fiberscope or other diagnostic imaging such as computed tomography. The clinical target volume (CTV) included the GTV and the entire vocal cords. The planning target volume (PTV) included the CTV plus a 0.5-cm margin in the anteroposterior axis, and a 0.5–1-cm margin in the craniocaudal axis.

All patients were treated with 4 or 6 MV photons by parallel-opposed bilateral fields with portal dimension of generally 6 × 6 cm centered over the thyroid cartilage. Elective irradiation to cervical lymph nodes was not performed. In this series, prescribed doses of RT were calculated at the central axis without heterogeneity correction.

The chemotherapeutic regimen consisted of daily low-dose CDDP plus weekly administration of DTX. CDDP (6 mg/m^2^, five times a week) was administered for up to 4 weeks (total dose of CDDP: 120 mg/m^2^) and DTX (10 mg/m^2^, once a week) was administered for up to four cycles from the commencement of RT in patients with adequate renal function, as determined by creatinine clearance (CCr). In the event of a fall in CCr below 60 mL/min, DTX alone (10 mg/m^2^, once a week) was administered for up to 4 weeks. RT was performed as soon as possible after the administration of chemotherapy. Granulocyte-colony stimulating factors were not used except for patients with febrile neutropenia or those with Grade 4 leukopenia or neutropenia. Patients who experienced hematological toxicity > Grade 3 were made to skip chemotherapy. Toxicity was assessed using the National Cancer Institute Common Toxicity Criteria for Adverse Events, version 4.0. Local response was assessed 1 month after the completion of RT on laryngeal fiberscope or by computed tomography scan of the head and neck region.

### Statistics

All statistical analyses were performed with IBM SPSS Statistics for Windows, version 21.0 (SPSS Inc., Armonk, NY, USA). The survival time was measured from the first day of treatment to the date of death or the date of most recent follow-up. The Kaplan–Meier method was employed to determine the actuarial survival rate and local control rate. Prognostic relevance of variables was assessed using log-rank test.

## Results

The median total irradiated dose was 66 Gy (range, 62–70 Gy), and the median overall duration of RT was 48 days (range, 43–52 days). Forty four patients completed the prescribed chemotherapy regimen (completion rate, 71%). In the remaining 18 patients, the reasons for interruption of chemotherapy included renal dysfunction (*n* = 15), grade 3 neutropenia (*n* = 1), liver dysfunction (*n* = 1), and drug eruption with oral mucositis (*n* = 1).

Median duration of follow up was 70 months (range, 8–139 months).

**Fig. 1:**
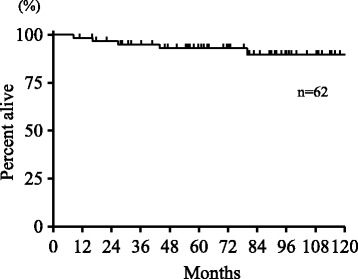
Overall survival from the start of treatment

**Fig. 2:**
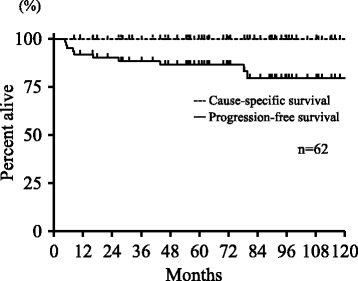
Cause-specific (solid dotted) and progression-free (solid line) survival from the start of treatment

The Kaplan–Meier overall survival (OS), cause-specific survival (CSS), and progression-free survival (PFS) curves from the initial treatment are shown in Figs. 1 and 2. The actuarial 3-year and 5-year OS rates were 95 and 93%, respectively. The 3-year and 5-year CSS rates were both 100%; the 3-year and 5-year PFS rates were 89 and 87%, respectively. Death from causes unrelated to glottic cancer were observed in five patients [cerebral infarction (*n* = 1), cerebral bleeding (*n* = 1), esophageal cancer (*n* = 1), lung cancer (*n* = 1), and pneumonia (*n* = 1)].

The initial local tumor response was evaluated in all patients. Fifty nine patients achieved a complete response while three patients showed a partial response and experienced disease progression 4 to 7 months after the start of treatment. Analysis of the failure patterns revealed progression only within the irradiation field in these three patients, and in additional two patients on extended follow up (78 months from the treatment). Progression outside the irradiation field was never observed as of the most-recent follow-up.

**Fig. 3:**
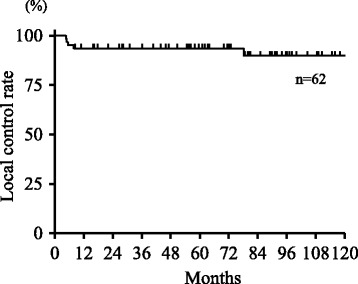
Local control rate during the follow-up period

The local control rate during the follow-up period is shown in Fig. 3. The actuarial 3-year and 5-year local control rates were both 94%. In these five patients with local recurrence, three patients completed the prescribed regimen of CCRT, while the other two patients could not complete chemotherapy because of renal dysfunction. Salvage surgery was performed in all five patients who experienced local recurrence. Four patients underwent total laryngectomy, one underwent larynx conserving surgery; all patients were salvaged from the disease. Another patient lost his larynx because of laryngeal necrosis after the treatment, so the actuarial 3-year and 5-year preservation rates of the larynx were both 94%, respectively. In a patient with late local recurrence (78 months after treatment), final pathological diagnosis at salvage surgery was adenosquamous carcinoma.

Hematologic and non-hematologic toxicities are summarized in Table 1. Among hematologic toxicities, neutoropenia of severity ≥ Grade 3 was observed in 8% of the patients. Of the non-hematologic toxicities, radiation mucositis of severity ≥ Grade 3 occurred in one patient (2%), while Grade 1/2 mucositis was noted in fifty patients (97%). Dermatitis of severity ≥ Grade 3 was observed in one patient, and this patient also experienced laryngeal necrosis.

**Table 1:** Treatment related adverse events

Toxicity	Grade					
	0	1	2	3	4	≥Grade 3 (%)
Leukopenia	26	16	17	3	0	5
Neutropenia	32	14	11	5	0	8
Anemia	28	31	3	0	0	0
Thrombocytopenia	33	27	2	0	0	0
Mucositis	1	15	45	1	0	2
Dermatitis	1	58	2	1	0	2
Hoarseness	45	12	4	1	0	2

## Discussion

Functional preservation is a key objective in treatment of head and neck malignancies. Comparable rates of local control, laryngeal voice preservation, ultimate local control, and survival were reported in patients with T1-T2 glottic cancer treated with transoral laser excision, open partial laryngectomy, and RT [15]. American Society of Clinical Oncology clinical practice guidelines recommend that patients with T1-T2 laryngeal cancer should be treated with the intent to preserve the larynx and that these patients can be treated with RT or larynx-preservation surgery with similar survival outcomes [16]. Some retrospective studies show approximately 70 to 80% 5-year local control with use of CFRT alone for T2 glottic lesions [1–6]. In this series, 62 patients with T2N0 glottic cancer who were treated by CCRT (RT and daily low-dose administration of CDDP plus weekly administration of DTX) had 5-year CSS, PFS, and local control rates of 100, 89, and 94%, respectively. These treatment results seemed to be comparable or superior to those of patients with T1 glottic cancer treated by RT alone [1–6].

High dose CDDP plus RT is recommended for the treatment of locally advanced head and neck cancers [17]. However, for the treatment of T2N0 glottic cancer, RT alone may achieve 70 to 80% local control and the purpose of concomitant use of chemotherapy is 10 to 15% gain by radiosensitization. It is conceivable that CCRT may be an overtreatment in some cases with T2 disease, but RT and daily low-dose administration of CDDP plus weekly administration of DTX regimen was well tolerated in some elderly patients, and 71% of all patients were able to complete the prescribed chemotherapy regimen. A completion rate of chemotherapy (71%) was not so high, but the priority of the treatment is the accomplishment of RT and the issue of safety, and the stopping rule of chemotherapy (concerning the use of cisplatin) was strict (The event of a fall in creatinine clearance below 60 mL/min, corresponsive to grade 1 adverse event). As for the other adverse events of CCRT, both radiation mucositis of severity ≥ Grade 3 and radiation dermatitis of severity ≥ Grade 3 occurred in 2% of all patients. One patient experienced laryngeal necrosis eight months after the initial treatment, for which total laryngectomy was performed. Grade 3 dermatitis was observed only in this patient, and chemotherapy was stopped midway through the prescribed regimen (total dose of RT was 64 Gy). The reason for severe adverse event in this patient is not clear, but this patient may have developed hypersensitivity to RT or chemotherapy.

Altered fractionation of RT schedule is one of the approaches used to improve tumor control. Garden performed a retrospective analysis of use of RT for T2 glottic cancer, and concluded that patients treated with HFRT (median dose: 77 Gy) showed improved local control from that of patients treated with CFRT [18]. Moreover, patients treated with AFRT (daily fraction sizes of 2.06–2.26 Gy) also showed improved local control from that achieved with CFRT (5-year local control rate: CFRT 59%, HFRT 79%, AFRT 82%). Motegi reported a 5-year local control rate of 77% in patients with early glottis cancer (T2) treated with AFRT (2.4 Gy once-daily fractionation) [19]. None of the patients experienced severe acute toxicity; while 2 out of 44 patients developed severe late toxicity (arytenoid edema). Thus, the trend and spotlight of treatment strategy of early stage glottic cancer has been a prospective randomized trial of CFRT versus HFRT/AFRT. Two recently reported prospective randomized trials in patients with glottic cancer showed no significant difference between CFRT and HFRT/AFRT [7, 8]. In the RTOG 9512 trial, the 5-year local control rate with HFRT (79.2 Gy in 66 fractions of 1.2 Gy administered twice a day) was modestly but not significantly higher than that with CFRT (78% vs. 70%; *P* = 0.14) [7]. KROG-0201 was a prospective randomized trial of AFRT (63 Gy/28 fractions for T1 and 67.5 Gy/30 fractions for T2) versus CFRT in patients with T1–2 glottic cancer. Of the enrolled patients, 89% had T1 disease. The 5-year local progression-free survival in the CFRT and AFRT arms was 77.8 and 88.5%, respectively (*P* = 0.213). A significant superiority of AFRT with respect to local control is yet to be demonstrated in randomized trials.

In T2 glottic cancer, chemoradiotherapy may achieve better tumor control than that achieved with RT alone. However, there are only a few reports from prospective randomized trials of chemoradiotherapy in these patients. Much of the available evidence emanates from retrospective studies. Itoh reported a 5-year local control rate of 91% with CCRT in a study of 11 patients with T2 glottic cancer [CFRT (total dose: 60 Gy) plus protracted continuous infusion of low-dose CDDP and 5-fluorouracil] [20].

Hirasawa et al. performed a retrospective multi-institutional analysis of 270 patients with T1–2 glottic cancer. The chemoradiotherapy group showed a tendency for superior local control in patients with T2 disease when compared with the RT group [21]. In this multi-institutional analysis, the regimen of chemotherapy varied in different treatment facilities. The 5-year local control rate was 66.4% for RT alone and 80.7% for chemoradiotherapy (*P* = 0.149).

Nakayama et al. reported the results of Phase I/II trial of concurrent use of orally administered S-1 and RT for T2 glottic cancer [22]. The use of S-1 at a daily dose of 80 mg/m^2^ with a 1-week break during the course of CFRT (60 Gy in 30 fractions) was reported to be safe and associated with a 3-year local control rate of 94.7%. The number of treated patients was relatively small (*n* = 19) and the findings need to be confirmed in a larger study. However, this is a rare report of prospective trial of CCRT for treatment of T2 glottic cancer.

In this series, the treatment outcomes in 62 consecutive patients who were treated by CCRT (RT and daily low-dose CDDP plus once-weekly DTX) were retrospectively investigated. The 5-year local control rate was 94%. A limitation of this regimen (weekly DTX and low-dose CDDP) is the need for hospitalization for drug and fluid infusion. Furusaka et al. reported the results of CCRT with once-weekly or twice-weekly DTX (10 mg/m^2^) with CFRT (total dose: 66 Gy). The 5-year laryngeal preservation rates were 83.8% in the once-weekly combination group and 97.6% in the twice-weekly combination group. The 5-year laryngeal preservation rate was 60.4% in the 57 patients who were treated with CFRT alone [23]. Once or twice weekly DTX may be administered in an outpatient setting, which is an advantage of CFRT with twice-weekly DTX.

## Conclusion

The feasibility and efficacy of CCRT with weekly DTX and low-dose CDDP for patients with T2N0 glottic cancer was confirmed in this series. The actuarial 3-year and 5-year OS rates were 95% and 93%, respectively. The actuarial 3-year and 5-year local control rates were both 94%, respectively. CCRT for T2 glottic cancer is not always fundamental in every facility; however, CCRT with weekly DTX and daily low-dose CDDP appears to be a safe and promising therapeutic modality and improvement in local control might be expected.

## Abbreviations

CCRT: Concurrent chemoradiotherapy
CDDP: Cisplatin
DTX: Docetaxel
RT: Radiation therapy
HFRT: Hyperfractionated radiotherapy
AFRT: Accelerated fractionated radiotherapy
CFRT: Conventional fractionated radiotherapy
GTV: Gross tumor volume
CTV: Clinical target volume
PTV: Planning target volume
CCr: Creatinine clearance
OS: Overall survival
CSS: Cause-specific survival
PFS: Progression-free survival
